# The Stockman’s Scorecard: quantitative evaluation of beef cattle stockmanship

**DOI:** 10.1093/tas/txaa175

**Published:** 2020-09-21

**Authors:** John K Yost, Jarred W Yates, Matt P Davis, Matthew E Wilson

**Affiliations:** 1 Davis College of Agriculture, Natural Resources, and Design, West Virginia University, Morgantown, WV; 2 Feedlot Services Division, Texas Cattlefeeders Association, Amarillo, TX

**Keywords:** cattle handling, stockmanship, welfare

## Abstract

An animal’s action, or inaction, is the direct result of a stockman’s action or inaction. The Stockman’s Scorecard is a novel observation instrument that has been proven to be a valid and reliable tool to measure the quality of beef cattle stockmanship. Specific handler actions have been weighted based on their perceived negative relationship to cattle stress from handling. The purpose of this article is to 1) document the initial use of the scorecard in a beef cattle feedlot setting and 2) provide further support to its validity by establishing an association with other quantitative and qualitative means of evaluating stockmanship. The Scorecard was used at 39 beef feedlots in Texas between March 2018 and April 2019. Eighty-four stockman were observed, and the average score received was 84.5 (SD = 14.73, range = 20 to 100). The most frequent mistakes observed were as follows: fills crowd pen/tub over half full (*n* = 39), slow to remove pressure (*n* = 29), uses unnecessary noise (*n* = 25), stands in front and taps rear (*n* = 24), and fails to regulate animal flow through a pinch point (*n* = 22). A strong negative association (ρ = −0.51) was found between the points deducted from the Noise and Physical Contact theme of the Scorecard and the number of animals touched with an electric prod from the BQA Feedyard Assessment. Moderate negative associations were found between the Scorecard final score and the number of animals that vocalize in the chute prior to procedures (ρ = −0.31). Those stockmen that scored above average on the Scorecard were qualitatively observed to be calm and quiet while working with the cattle (Kappa = 0.44). The qualitative disposition of cattle had little effect on the final score of stockmen using the Scorecard (Kappa = 0.17). The use of the Scorecard in a feedlot setting has demonstrated that as stockman scores decrease, there is an increase in the number of negative actions toward cattle and a negative behavioral response of the cattle themselves. Establishment of an association between a stockman’s score using the Stockman’s Scorecard and the animal-based observations from the BQA Feedyard Assessment further strengthens the validity of the Stockman’s Scorecard as a tool to measure the quality of beef cattle stockmanship. The Scorecard has application as a tool to identify specific stockmanship deficiencies in order to target stockmanship training.

## INTRODUCTION

The behavior and actions of stockmen has a direct effect on the behavior and welfare of livestock (Zulkifli, 2013). The result of this human–livestock interaction is dependent on the attitudes and behavior of the stockperson (Waiblinger et al., 2002). Behavioral research in beef cattle (Petherick et al., 2009a; Probst et al., 2013), dairy cattle (Rushen et al., 1998; Waiblinger et al., 2003), and swine (Tallet et al., 2014) has shown that an animal’s response is dependent on the quality of treatment received from their human handlers. In dairy cattle (Passille et al., 1996; Munksgaard et al., 1997), beef cattle (Boivin et al., 1998), and sheep (Boivin et al., 1997), there is support that livestock may be able to differentiate between handlers based on their familiarity with the stockman and the quality of the stockperson’s handling. Also, it has been shown in pigs that group behavior was altered when a single pen mate was subjected to negative handling practices although the others of the group did not receive the treatment (Reimert et al., 2017). Beef cattle will habituate to common handling practices and human contact by frequent exposure (Matson, 2006), especially at a younger age (Fukasawa, 2012; Etim et al., 2013). However, livestock will not habituate to painful procedures and adverse handling practices (Grandin et al., 1986).

Livestock handling involves both the restraint of animals, and encouraging a desired movement, in a way that minimizes fearful reactions (Gonyou, 1995). Stockmen are encouraged to be calm, quiet, slow, and deliberate when working animals (Grandin, 2015, 65–95). Furthermore, stockmen need to understand the behaviors of cattle, and their physiology, in order to take advantage of their natural prey instinct when herding (Grandin and Dessing, 2008). Evaluation of stockmanship is a critical component in assuring positive animal welfare (Grandin, 2001; Grandin, 2014). Assessment of stockmanship involves the observation of animal behaviors and quantitative measurements of their temperament. Chute scoring, chute exit speed scoring, vocalization tests, and aversion tests are all measures to evaluate the overall treatment of cattle (Grandin, 1994; Grandin and Shivley, 2015). The livestock industry has been proactive in assessing the care of livestock at the farm and processing levels through facility evaluations such as the BQA Feedyard Assessment (2017), the North American Meat Institute Audit (2019), and the European Welfare Quality Audit (Welfare Quality Network, 2009). As general themes, the assessments seek to discover whether appropriate management protocols are in place to insure the implementation of scientifically based, industry-recognized, best management practices. Within these evaluations, highly reliable, animal-based measurements are utilized to determine the quality of stockmanship (Grandin, 2015, 69 to 95). Specifically, the BQA Feedyard Assessment asks that 100 head of cattle be observed to determine the number of cattle that are touched with an electric prod, fall upon release from the chute, stumble/trip when released from the chute, vocalize in chute before procedures, jump or run when released from the chute, or miscaught and not readjusted while in the chute.

Although these measurements are appropriate to assess improvements in stockmanship within an operation (Rushen and De Passille, 2015), how are we to determine what stockperson actions caused any aberrations identified in these animal observations? The argument has been made that the human factor may strongly influence audit results (Rocha et al., 2016). Coleman and Hemsworth (2014) are quoted as saying, “While welfare monitoring schemes are likely to improve animal welfare, the impact of such schemes will only be realized by recognizing the limitations of stockpeople, monitoring stockmanship and providing specific stockpersons training to target key aspects of stockmanship.” Gonyou (1995) stated, “The most important part of a livestock handling system are the persons who handle the animals and operate the facilities and equipment”. He goes on to say, “the potential of well-designed facilities and equipment will only be realized if the stockpersons use them properly.”

The Stockman’s Scorecard is an evaluation instrument designed to measure the quality of beef cattle stockmanship. The scorecard has previously been proven to be a valid and reliable tool for assigning a numerical score to the stockmanship abilities of cattle handlers (Yost et al., 2020). The card provides 30 observation points, identified from other published works, which can be interpreted as producing either a positive or negative animal behavior outcome. The observation points have been grouped into three categories identified as Situational Awareness, Herding Skill, and Noise/Physical Contact. The Situational Awareness category includes 13 observation points meant to assess if the stockman can function as a member of the animal handling team, if they know the capabilities of the handling facility to properly move animals through gate openings and other pinch points, if they know the proper number of animals to load into the crowd tub, and if they avoid attempting to work in the animal’s blindspot. The Herding Skill category groups seven observation points that seek to evaluate whether the handler understands how to use an animal’s flight zone, and point of balance, to produce positive animal movement. The Noise/Physical Contact section contains 10 observation points that evaluate the handler’s use of vocal and artificial noise during the handling activity, as well as if the handler is properly using electric prods or physical force to encourage animal movement. Each stockman begins an evaluation with 100 points. The observer deducts the specified points for each negative action performed by the subject. At the end of the evaluation, the total deductions are determined and subtracted from 100 to establish a final score. The purpose of this paper is to 1) document the initial use of the scorecard in a feedlot setting and 2) provide further support to its validity by establishing an association with other quantitative and qualitative means of evaluating stockmanship.

## MATERIALS AND METHODS

An Institutional Animal Care and Use Committee protocol was not required for this study. Three assessment tools were utilized for this study. They are as follows: the Stockman’s Scorecard (Yost et al., 2020); the BQA Feedyard Assessment (2017), which has six handling measures; and a qualitative scale describing the animals’ and handlers’ dispositions. To develop the qualitative scale, the observer was asked to use their own words to briefly describe the disposition of the cattle and the stockman. For a stockman, they could use words such as calm, angry, hurried, or nervous. For the cattle, they could use words such as calm, stubborn, flighty, or riled up. The handler and livestock disposition determinations were qualitatively evaluated by the researcher and condensed into themes. The themes were then coded to create a disposition scale. The coding for the stockman scale was as follows: 1 = calm/quiet, 2 = calm plus another descriptor, 3 = fast/rushed/excited, 4 = nervous/unsure/frustrated. The coding for the livestock scale was as follows: 1 = calm/quiet, 2 = slightly jumpy, 3 = excited/jumpy/wound-up, 4 = stubborn/hesitant. For analysis, codes were combined to create a nominal variable scale (handler, 1 = calm/quiet, 0 = other descriptor; livestock, 1 = calm/quiet, 0 = other descriptor), and Kappa was calculated with JMP (ver. 25) to determine the level of agreement between Scorecard score and the handler and livestock disposition scales.

Data collection was conducted through a cooperation with the Texas Cattlefeeders Association, Feedyard Services Division. Division personnel regularly conduct BQA Feedyard Assessments for member feedyards, and each employee conducting the audits has been certified by the Professional Animal Auditor Certification Organization (PAACO, paaco.org). All feedyards used in this study agreed to the use of the Stockman’s Scorecard during a normally scheduled Assessment. The observers were provided with an observation instrument that included the Stockman’s Scorecard and the animal-based observations recording component of the BQA Feedyard Assessment. Prior to any data collection, the observers were provided a narrated PowerPoint presentation that detailed the methodology of the scorecard and its use. The PowerPoint presentation provided an explanation of each observation point included on the Scorecard, and examples of situations that represent the inclusion of the observation point. Once the materials had been reviewed, a conference call was held with the primary researcher and the observers to explain the intent of the evaluation, the desired data to be collected, and to answer any questions or provide clarity on the methodology and use of the card.

Data collection occurred over the period of 1 yr (March 2018 to April 2019). The Scorecard was used to evaluate 86 stockmen from 39 cattle feedyards in Texas. Nine facilities were visited once, 19 facilities were visited twice, 6 facilities were visited three times, 4 facilities were visited four times, and 1 facility was visited five times. All subjects evaluated were stationed between the crowd pen/tub and the chute. The observers were asked to evaluate one to two employees at each facility, but not the same employee if the facility was visited on multiple occasions, using the scorecard as they were conducting a normally scheduled BQA Feedyard Assessment. The observers evaluated each subject using the scorecard criteria and collected the animal observation data on a maximum of 100 head through the handling system.

Completed scorecards were scanned by computer and stored as PDF files to be emailed to the researcher. Once received, the individual scorecard results were entered into an Excel spreadsheet. The data for each observation point were recorded as a “zero” or a “1.” If an action, on the part of the stockman, was observed, it was recorded as a “1.” All unobserved observation points were recorded as a “zero.” Frequencies and standard deviations were determined by analysis with Microsoft Excel. Spearman’s rho correlation to determine the associations between the Scorecard, and BQA Feedyard Assessment results were performed with JMP (ver. 25). For the Spearman’s correlation analysis, a Benjamini–Hochberg adjustment was used with a 10% false discovery rate used in the calculation. Statistical significance was set a priori at α = 0.05.

## RESULTS AND DISCUSSION

### Quantitative Evaluation of Stockmanship

**Figure 1.**
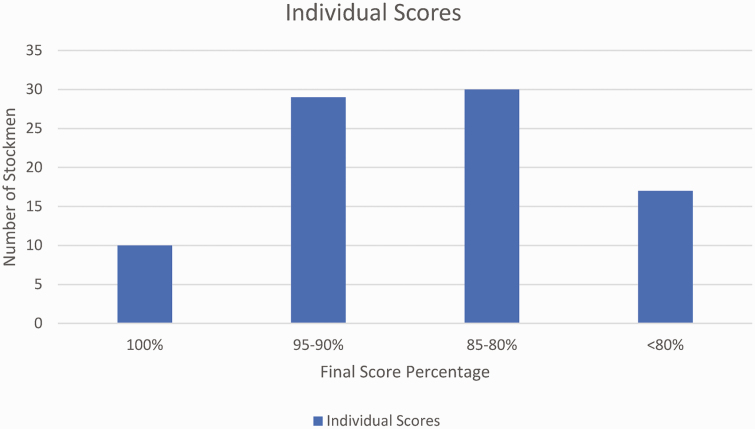
Individual scores using the Stockman’s Scorecard.

The average Stockman’s Scorecard score received was 84.5 (SD = 14.73, range = 100 to 20). Forty-five percent of the stockmen observed (*n* = 39) received a perfect score or were documented to have performed one to two actions that would deduct points (Figure 1). The most frequent mistakes observed were as follows: fills crowd pen/tub over half full (*n* = 29), slow to add/remove pressure (*n* = 27), uses unnecessary noise (*n* = 25), stands in front of the animal and taps on rear (*n* = 24), and fails to regulate animal flow through a pinch point (*n* = 22; Table 1). In addition, other common mistakes were when the stockmen unintentionally worked in an animal’s blindspot (*n* = 18) and were observed to be constantly, and unnecessarily, screaming or yelling at the cattle (*n* = 13).

**Table 1.** Stockman’s Scorecard results

Observation point	Points deducted	Number observed	Percent observed
Valued team contributor	0	78	91
Operates independent of team	−10	0	0
Ineffective team member	−5	8	9
Fills crowd pen ½ full or less	0	52	60
Over fills crowd pen	−10	2	2
Fills crowd pen over ½ full	−5	29	34
Regulates cattle flow through pinch point	0	58	67
Forces cattle through pinch point	−10	4	5
Fails to regulate cattle flow through pinch point	−5	22	26
Avoids working in cattle blindspot	0	64	74
Continually works in blindspot	−10	3	3
Unintentionally works in blindspot	−5	18	21
Understands cattle’s point of balance	0	57	66
No understanding of point of balance	−10	5	6
Stands in front of animal/taps rear	−5	24	28
Effectively uses flight zone pressure	0	55	64
Excessive flight zone pressure	−10	3	3
Slow to add/remove pressure	−5	27	31
Unable to move group as a unit	−5	2	2
Uses appropriate amount of noise	0	45	52
Intentionally generates metallic noise	−10	7	8
Constant/unnecessary screaming/yelling	−10	13	15
Unnecessary noise	−5	25	29
Driving aid used appropriately	0	69	80
Electric prod primary driving aid	−10	7	8
Electric prod applied at wrong time	−5	11	13
Uses appropriate physical contact	0	80	93
Excessive/unnecessary physical contact	−10	6	7
Tail twisting after animal movement	−5	0	0

In other studies that have documented stockman actions toward beef cattle, there has been a high level of variability between operations and individual stockmen (Hultgren et al., 2013; Ligon, 2014; Simon et al., 2016; Destrez et al., 2018). In all cases, cattle that were subjected to increased intensity of human vocalization and physical contact were also perceived as more difficult to move through the handling system. Beef cattle stockman should make a conscientious effort to handle cattle in a way that stress is minimized. Aversive handling practices induce significant fear in cattle, which can cause serious losses in productivity, increased handling problems and related injuries to both animals and handlers, and diminished animal welfare (Rushen et al., 1999). Specific cattle handling recommendations have been provided in published research (Grandin, 2008; North American Meat Institute, 2019; Grandin, 2015). Elevated stress has been shown to be caused when handlers scream and yell, crack whips, generate metallic noise by banging on gates, run at the animal, and aggressively hit cattle (Waynert et al., 1999; Grandin, 2008; Woiwode et al., 2016a).

**Figure 2.**
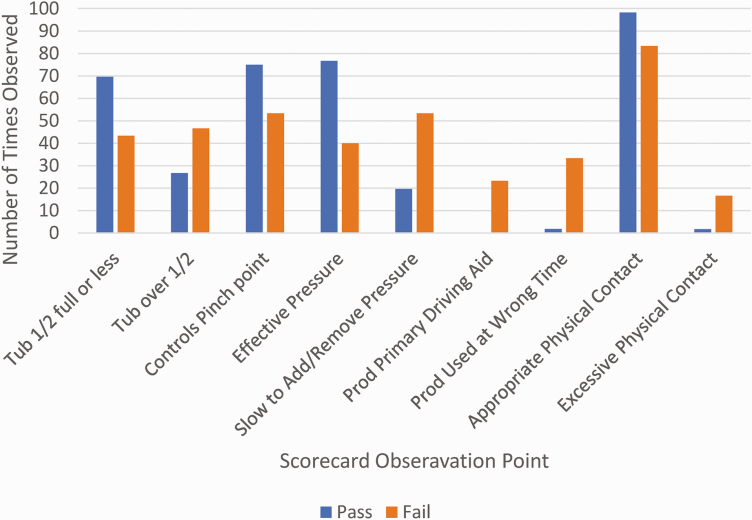
Comparison of Stockman score for facilities that “passed” or “failed” a category of the BQA Feedyard Assessment cattle handling component.

Stockmanship assessments were also conducted for the facility during scheduled BQA Feedyard Assessments. The Assessment uses six animal-based observations to determine the quality of stockmanship. For each observation point, thresholds have been established to determine whether the facility “passes” or “fails” on cattle handling (Figure 2). Of the 39 facilities visited, 24 (61%) failed on one or more categories, on one or more visits. These 24 yards were visited a total of 53 times during the sampling period, and there were 30 documented failures. Six of these facilities were only sampled once, two feedyards failed on all visits, and the remaining 16 passed on at least one of their other sampling dates. The most frequent cause of a failure was the use of electric prods (20%), stumble/tripped when released from the chute (9%), and miscaught in the head chute and not readjusted (6%; Table 2). The number of facilities that failed our animal handling assessment is higher than other reported observations (Barnhardt, 2015; Woiwode et al., 2016b). The differences may be due to the fact that most of the yards we sampled were visited multiple times during the study period, instead of a single observation as in the other studies.

**Table 2.** BQA Feedyard Assessment animal observation category fails by individual feedyards

Observation category	BQA pass threshold %	Number of fails	Percent fails
Electric prod use	<10.0	15	20
Fell when released from chute	<2.0	1	1
Stumble/tripped when released from chute	<10.0	8	9
Vocalized in chute prior to procedures	<5.0	3	3
Jumped/tan when released from chute	<25.0	1	1
Miscaught in head chute and not readjusted	0.0	5	6
Single-category fail		15	
Two-category fail		5	

The average Scorecard score for facilities that passed the BQA Feedyard Assessment was 90.0, whereas the facilities that failed on the animal handling component received a final score of 74.3 (*P* < 0.0001). For those facilities that failed the handling portion of the assessment, the most frequent mistakes observed were as follows: fills crowd tub over ½ full, slow to add remove pressure, used electric prod as the primary driving aid, applied the use of an electric prod at the wrong time, and used excessive physical contact. Likewise, for those facilities that passed the animal component of the assessment, the employees were observed to fill the tub ½ full or less, regulated the flow of animals through a pinch point, demonstrated effective use of flight zone pressure, and utilized appropriate physical contact.

Several negative associations were found between a subject’s score on the Scorecard and the animal-based measurements collected with the BQA Feedyard Assessment (Table 3). A strong negative association (Robinson et al., 1991) was found between the number of animals touched with an electric prod and the subjects score on the noise and physical contact section (ρ = −0.51). This high association should be expected as both tools collect a similar measurement. Points are lost in the noise and physical contact theme and deducted from the stockman’s final score if an electric prod is used excessively or if contact is applied at the wrong time. The Assessment asks the observer to count the number of animals that are touched with the prod. Moderate negative associations (Robinson et al., 1991) were found between the use of electric prods and the Situational Awareness (ρ = 0.31) score and Final Score (ρ = −0.43) on the Scorecard. Also, moderate negative associations were found between the number of animals that vocalize in the chute prior to procedures and the final score (ρ = −0.31) and herding skill (ρ = −0.31) section on the scorecard. Grandin (1998) has identified animal vocalization as a key indicator of stress from adverse handling practices. She observed that skilled handlers averaged 4.5% animal vocalizations where plants with aggressive handling approached 22%.

**Table 3.** Associations between the Scorecard results and the BQA Feedyard Assessment animal observation categories

Association	Spearman ρ	*P*-value	Strength of association
BQA 1^1^ vs. NP Total^3^	−0.51	<0.0001	Strong
BQA 1^1^ vs. Final Score^6^	−0.43	<0.0001	Moderate
BQA 1^1^ vs. SA Total^4^	−0.31	0.0038	Moderate
BQA 4^2^ vs. Final Score^6^	−0.31	0.0041	Moderate
BQA 4^2^ vs. HS Total^5^	−0.31	0.0041	Moderate

^1^BQA Feedyard Assessment measurement for frequency of electric prod use on 100 head of cattle.

^2^BQA Feedyard Assessment measurement for frequency of cattle vocalizing in the chute, following restraint but prior to occurrence of a procedure, per 100 head.

^3^Sum of points deducted on scorecard from observation points under the Noise and Physical Contact theme.

^4^Sum of points deducted on scorecard from observation points under the Situational Awareness theme.

^5^Sum of points deducted on scorecard from observation points under the Herding Skill theme.

^6^Final score on scorecard received by subtracting all deductions from 100 possible points.

### Qualitative Description of Stockman and Livestock Disposition

The observers were asked to provide a one word, or short phrase, description of the handler’s and the livestocks’ disposition. The majority of stockmen were described as being calm (*n* = 60; Table 4). There was an additional seven stockmen that were described as calm, but the observer also documented that they seemed rushed or were noisy. On 15 evaluations, the handlers were only described as being noisy, rushed, excited, jumpy, nervous, or frustrated. When describing the cattle being processed, 30% of the groups were categorized as being calm, whereas many groups were observed to be “slightly jumpy” (*n* = 16) or “excited/wound up” (*n* = 34). A small number of the groups (*n* = 6), usually Holstein cattle, were described as being “stubborn.”

**Table 4.** Frequency of qualitative descriptions for handler and livestock disposition

	Number observed	Percent observed
Handler disposition		
Calm	60	70
Calm but rushed or noisy	7	8
Noisy	3	3
Rushed	5	6
Excited	4	5
Jumpy/nervous/frustrated	3	3
Cattle disposition		
Calm	26	30
Slightly jumpy	16	19
Excited/wound up	34	37
Stubborn	6	7

There was a moderate level of agreement (Stokes et al., 1995) between the qualitative description of the stockman’s behavior and their final score using the Scorecard (Kappa = 0.44, *P* < 0.0001). Those stockmen that were observed to be calm in their actions tended to have a higher final score than those that were described as noisy, rushed, jumpy, nervous, or frustrated. A very slight agreement (Stokes et al., 1995) was found between the stockman’s final score and the livestock disposition descriptor (Kappa = 0.18, *P* = 0.01). In 43.9% of the cases where the livestock were described to have a negative disposition, the stockman scored high on the scorecard, and in 3.6 % of the cases, the livestock were described as “calm,” but the stockman received a low score.

Significant correlations have been found between stockman behavior, animal behavior, and animal productivity (Hemsworth et al., 2002; Waiblinger et al., 2002; Ellingsen et al., 2014). Livestock that are handled in a calm manner tend to behave calmer and have higher productivity than those that are handled more aggressively. We observed that there was a negligible association between handler score or disposition and animal behavior. The expressed behavior of cattle is related to a combination of environment, genetics, and handling factors (Grandin, 1994; Grignard et al., 2001). Cattle may initially react negatively to any handling practice but can habituate over time (Petherick et al., 2009a,b), although they will not habituate to extremely adverse handling practices (Grandin et al., 1986). We were not able to observe every stockman involved in the handling activity, nor did we collect data on the age of the cattle and their time at the feeding facility. Repeated interactions with humans have shown to reduce reactivity of cattle in a feedlot setting (Doyle, 2014). It is also believed that cattle can differentiate between handlers that treat them poorly and handlers that are gentle (Munksgaard et al., 1997).

## Conclusions

In order for an evaluation tool to be useful to measure the underlying construct it needs to be determined if it is valid and reliable. The Stockman’s Scorecard has been previously determined to be both valid and reliable in measuring the quality of stockmanship. This article has further strengthened the tool by establishing the criterion-related validity of the instrument (Huck, 2012, 83). To establish this type of validity, the new instrument is compared with current accepted measurement tools. The established associations between Scorecard’s results and animal-based observations from the BQA Feedyard Assessment provide the criterion-related validity. Furthermore, we have been able to provide an association between an individual score and the stockman’s behavior. The slight associate of the Scorecard results with a simple qualitative description of the cattle’s behavior implies that the score received by the individual stockman was independent of the behavior of the livestock.

The BQA Feedyard Assessment is a proven, reliable instrument used to evaluate stockmanship at the facility level. It allows managers to see the progress of stockman training to reduce animal stress and increase operational efficiency. The Stockman’s Scorecard now provides an additional resource to reinforce gains made through livestock handling training programs or to determine who may be the cause of deficiencies and establish targeted training programs to improve a handler’s stockmanship. This tool has multiple applications. It may be used in a pretest/post-test format for educators to evaluate stockmanship training. It can be used by researchers to precisely define the stockmanship parameters of their animal handling studies. Future research should focus on evaluation of all stockmen involved in an animal handling activity to determine whether a specific stockman can be identified as the cause of handling aberrations. There is also the opportunity to begin to determine the physiological effects of precise adverse handling conditions on animal outcomes.

## References

[CIT0001] Beef Quality Assurance Feedyard Assessment Guide (2017). https://www.bqa.org/Media/BQA/Docs/feedyard_assessment_2017.pdf.

[CIT0002] Barnhardt T R 2015 Implementation of industry-oriented welfare quality assurance assessment tools in commercial cattle feeding operations. Masters Thesis. Kansas State University.

[CIT0003] Boivin X, Garel J P, Mante A, and Neindre P L. 1998 Beef calves react differently to different handlers according to the test situation and their previous interactions with their caretaker. Appl. Anim. Behav. Sci. 55:245–257. doi:10.1016/S0168-1591(97)00050-6

[CIT0004] Boivin X, Nowak R, Desprès G, Tournadre H, and Le Neindre P. 1997 Discrimination between shepherds by lambs reared under artificial conditions. J. Anim. Sci. 75:2892–2898. doi:10.2527/1997.75112892x 9374301

[CIT0005] Coleman G L, and Hemsworth P H. 2014 Training to improve stockperson beliefs and behavior towards livestock enhances welfare and productivity. Rev. Sci. Tech. Off. Int. Epiz. 1:131–137. 10.20506/rst.33.1.2257 25000785

[CIT0006] Destrez A, Haslin E, and Boivin X. 2018 What stockperson behavior during weighing reveals about the relationship between humans and suckling beef cattle: A preliminary study. Appl. Anim. Behav. Sci. 209:8–13. doi:10.1016/j.applanim.2018.10.001

[CIT0007] Doyle R 2014 Don’t fence me in? The welfare of cattle in feedlots 2014 Graham Centre Beef Forum. https://www.csu.edu.au/_data/assets/pdf_file/0007/1180384/2014-Beef-Forum-Proceedings-WEB.pdf#page=23.

[CIT0008] Ellingsen K, Coleman G K, Lund V, and Meidell C M. 2014 Using qualitative behavior assessment to explore the link between stockperson behavior and dairy calf behavior. Appl. Anim. Behav. Sci. 153:10–17. doi:10.1016/j.applanim.2014.01.011

[CIT0009] Etim N N, Offiong E E A, Udo M D, Williams M E, and Evans E I. 2013 Physiological relationship between stress and reproductive efficiency. Agric. Biol. J. North Am. 4:600–604. doi:10.5251/anjna.2013.4.6.600.604

[CIT0010] Fukasawa M 2012 Calf training for loading onto vehicle at weaning. Anim. Sci. J. 83:759–766. doi:10.1111/j.1740-0929.2012.01020.x 23126329

[CIT0011] Gonyou H W 1995 How animal handling influences animal behavior. 1995 Annual Research Report. Prairie Swine Centre, Inc http://www.prairieswine.com/pdf/2007.pdf.

[CIT0012] Grandin T 1994 Solving livestock handling problems. www.grandin.com/references/solv.lvstk.probs.html.

[CIT0013] Grandin T 1998 The feasibility of using scoring as an indicator of poor welfare during cattle slaughter. Appl. Anim. Behav. Sci. 56:121–128. doi:10.1016/S0168-1591(97)00102-0

[CIT0014] Grandin T 2001 Livestock-handling quality assurance. J. Anim. Sci. 79(E suppl.):E239–E248. doi:10.2527/jas2001.79E-SupplE218x

[CIT0015] Grandin T 2014 Animal welfare and society concerns finding the missing link. Meat Sci. 98:461–469. doi:10.1016/j.meatsci.2014.05.011 24928166

[CIT0016] Grandin T 2015 How to improve livestock handling and reduce stress. In: Grandin T, editor, Improving animal welfare: A practical approach. 2nd ed CAB International, Wallingford, UK p. 69–95.

[CIT0017] Grandin T, and Dessing M. 2008 Human livestock handling: Understanding livestock behavior and building facilities for healthier animals. Storey Publishing, North Adams, MA p. 52–58.

[CIT0018] Grandin T, Curtis S E, Widowski T M, and Thurmon J C. 1986 Electro-immobilization versus mechanical restraint in an avoid-avoid choice test for ewes. J. Anim. Sci. 62:1469–1480. doi:10.2527/jas1986.6261469x 3733556

[CIT0019] Grandin T, Oldfield J E, and Boyd L J. 1998 Reducing handling stress improves both productivity and welfare. Prof. Anim. Sci. 14:1–10. doi:10.15232/S1080-7446(15)31783-6

[CIT0020] Grandin T, and Shivley C. 2015 How farm animals react and perceive stressful situations such as handling, restraint, and transport. Animals (Basel) 5:1233–1251. doi:10.3390/ani5040409 26633523 PMC4693213

[CIT0021] Grignard L, Boivin X, Boissy A, and Le Neindre P. 2001 Do beef cattle react consistently to different handling situations? Appl. Anim. Behav. Sci. 71:263–276. doi:10.1016/s0168-1591(00)00187-8. 11248377

[CIT0022] Hemsworth P H, Coleman G J, Barnett J L, Borg S, and Dowling S. 2002 The effects of cognitive behavioral intervention on the attitude and behavior of stockpersons and the behavior and productivity of commercial dairy cows. J. Anim. Sci. 80:68–78. doi:10.2527/2002.80168x 11831530

[CIT0023] Huck S W 2012 Reading statistics and research. 6th ed Pearson Education, Inc, Boston, MA.

[CIT0024] Hultgren J, Wiberg S, Berg C, Cvek K, and Kolstrup C. 2013 Cattle behaviors and stockperson actions related to impaired animal welfare at Swedish slaughter plants. Appl. Anim. Behav. Sci. 152:23–37. doi:10.1016/j.applanim.2013.12.005

[CIT0025] Ligon J M 2014 The effects of low stress cattle handling and weaning training on post-weaning weight gain and calf activity. Master Thesis, Va Tech, Blacksburg, VA https://vtechworks.lib.vt.edu/bitstream/handle/10919/51262/Ligon_JM_T_2015.pdf?sequence=1&isAllowed=y.

[CIT0026] Matson K M 2006 The effect of weekly handling on the temperament of peripuberal crossbred beef heifers. Masters Thesis. Animal and Poultry Sciences, Virginia Tech.

[CIT0027] Munksgaard L, De Passille A M, Rushen J, Thodberg K, and Jensen M B. 1997 Discrimination of people by dairy cows based on handling. J. Dairy Sci. 80:1106–1112. doi:10.3168/jds.S0022-0302(97)76036-3 9201580

[CIT0028] North American Meat Institute. 2019 Recommended animal handling guidelines and audit guide: A systematic approach to animal welfare. http://www.animalhandling.org/ht/d/sp/i/26752/pid/26752.

[CIT0029] Passille A M, Rushen J, Ladewig J, and Petherick C. 1996 Dairy calves’ discrimination of people based on previous handling. J. Anim. Sci. 74:969–974. doi:10.2527/1996.745969x 8726728

[CIT0030] Petherick J C, Doogan V J, Venus B K, Holroyd R G, and Olsson P. 2009b Quality of handling and holding yard environment, and beef cattle temperament: 2. Consequences for stress and productivity. Appl. Anim. Behav. Sci. 120:28–38. doi:10.1016/j.applanim.2009.05.009

[CIT0031] Petherick J C, Doogan V J, Holroyd R G, Olsson P, and Venus B K. 2009a Quality of handling and holding yard environment, and beef cattle temperament: 1. Relationships with flight speed and fear of humans. Appl. Anim. Behav. Sci. 120:18–27. doi:10.1016/j.applanim.2009.05.008

[CIT0032] Probst J K, Hillmann E, Leiber F, Kreuzer M, and Neff S N. 2013 Influence of gentle touching applied few weeks before slaughter on avoidance distance and slaughter stress in finishing cattle. Appl. Anim. Behav. Sci. 144:14–21. doi:10.1016/j.applanim.2012.12.007

[CIT0033] Reimert I, Fong S, Rodenburg T B, and Bolhuis J E. 2017 Emotional states and emotional contagion in pigs after exposure to a positive and negative treatment. Appl. Anim. Behav. Sci. 193:37–42. doi:10.1016/j.applanim.2017.03.009

[CIT0034] Robinson J P, Shaver P R, and Wrightsman L S. 1991 Criteria for scale selection and evaluation. In: Robinson J P, Shaver P R, and Wrightsman L S, editors, Measures of personality and social psychological attitudes. Academic Press, New York, NY p. 1–16.

[CIT0035] Rocha L M, Velarde A, Dalmau A, Saucier L, and Faucitano L. 2016 Can the monitoring of animal welfare parameters predict pork meat quality variation through the supply chain (from farm to slaughter)? J. Anim. Sci. 94:359–376. doi:10.2527/jas.2015-9176 26812341

[CIT0036] Rushen J and De Passille A M. 2015 The importance of good stockmanship and its benefits to animals. In: Grandin T, editor, Improving animal welfare: A practical approach. 2nd ed. CAB International, Wallingford, UK p. 125–138.

[CIT0037] Rushen J, Munksgaard L, Passille A M B, Jensen M B, and Thodberg K. 1998 Location of handling and dairy cows’ responses to people. Appl. Anim. Behav. Sci. 55:259–267. doi:10.1016/S0168-1591(97)00053-1

[CIT0038] Rushen J, Taylor A, and Passille A M D. 1999 Domestic animals’ fear of humans and its effect on their welfare. Appl. Anim. Behav. Sci. 65:285–303. doi:10.1016/S0168-1591(99)00089-1

[CIT0039] Simon G E, Hoar B R, and Tucker C B. 2016 Assessing cow-calf welfare. Part 1: Benchmarking beef cow health and behavior, handling; and management, facilities, and producer prospectives. J. Anim. Sci. 94:3476–3487. doi:10.2527/jas2016-0308 27695797

[CIT0040] Stokes M E, Davis C S, and Koch G G. 1995 Categorical data analysis using the SAS system. SAS Institute, Inc, Cary, NC.

[CIT0041] Tallet C, Kardiatou S, Prunier A, Noward R, Boissy A, and Boivin X. 2014 Behavioural and physiological reactions of piglets to gentle tactile interactions vary according to their previous experience with humans. Livest. Sci. 167:331–341. doi:10.1016/j.livsci.2014.06.025

[CIT0042] Waiblinger S, Menke C, and Coleman G. 2002 The relationship between attitudes, personal characteristics and behavior of stockpeople and subsequent behavior and production of dairy cows. Appl. Anim. Behav. Sci. 79:195–219. doi:10.1016/S0168-1591(02)00155-7

[CIT0043] Waiblinger S, Menke C, and Folsch D W. 2003 Influences on the avoidance and approach behavior of dairy cows towards humans on 35 farms. Appl. Anim. Behav. Sci. 84:23–39. doi:10.1016/S0168-1591(03)00148-5

[CIT0044] Waynert D F, Stookey J M, Schartzkopt-Genswein K S, Watts J M, and Waltz C S. 1999 The response of beef cattle to noise during handling. Appl. Anim. Behav. Sci. 62:27–43. doi:10.1016/S0168-1591(98)00211-1

[CIT0045] Welfare Quality. 2009 Welfare Quality® assessment protocol for cattle. Welfare Quality Consortium, Lelystad, The Netherlands.

[CIT0046] Woiwode R, Grandin T, Kirch B, and Paterson J. 2016a Effects of initial handling practices on behavior and average daily gain of fed steers. Int. J. Livest. Prod. 7:12–18. doi:10.5897/IJLP2015.0277

[CIT0047] Woiwode R, Grandin T, Kirch B, and Paterson J. 2016b Compliance of large feedyards in the northern high plains with the Beef Quality Assurance Feedyard Assessment. Prof. Anim. Sci. 32:750–757. doi:10.15232/pas.2015-01472

[CIT0048] Yost J K, Yates J W, Davis M P, and Wilson M E. 2020 The Stockman’s Scorecard: Validity and reliability as an instrument for measuring stockmanship. J. Extension. 58 https://joe.org/joe/2020april/tt4.php.

[CIT0049] Zulkifli I 2013 Review of human-animal interactions and their impact on animal productivity and welfare. J. Anim. Sci. Biotechnol. 4:25. doi:10.1186/2049-1891-4-25 23855920 PMC3720231

